# Optimising Conventional Therapy in Rheumatoid Arthritis

**DOI:** 10.31138/mjr.311025.eti

**Published:** 2026-03-01

**Authors:** Alexandros A. Drosos, Paraskevi V. Voulgari

**Affiliations:** Department of Rheumatology, School of Health Sciences, Faculty of Medicine, University of Ioannina, Ioannina, Greece

**Keywords:** rheumatoid arthritis, methotrexate, antirheumatic agents, biologic therapies

## Abstract

In the decades of the 1980s and 1990s, before the onset of biologic therapies for rheumatoid arthritis (RA) management, there was a significant number of patients who did not achieve remission or low disease activity (LDA), due to limited therapeutic choices. At that time, only some conventional synthetic disease modifying anti-rheumatic drugs (csDMARDs), along with steroids, were used. Nowadays, despite the progress of RA management with the introduction of biologic (b) and targeted synthetic (ts) DMARDs, which showed significant clinical improvement and retardation of radiological damage, unmet needs of RA treatment still exist. On the other hand, the concept of treat-to-target (T2T) approach and tight control monitoring of disease activity, are considered now the cornerstone for achieving remission or LDA in RA patients. Several clinical trials confirmed the efficacy and safety of csDMARDs using the tight control and T2T strategies. Methotrexate is the fundamental drug for RA management and in combination with other csDMARDs, with or without steroids, it has proven to be efficacious and safe and less expensive in comparison to newer biologic therapies. Furthermore, MTX demonstrated cardioprotective effects and reduced the risk of cardiovascular events in RA patients Therefore, there is a potential for improving treatment strategies with conventional therapies in the management of early RA. Optimal and early use of csDMARDs controls disease activity similarly to biologic therapies and is less expensive.

## INTRODUCTION

Rheumatoid arthritis (RA) is a chronic autoimmune inflammatory disease affecting the synovial membrane causing pain and inflammation, leading to erosions of the joints and bone destruction.^1,2^ It affects 0,5–1% of the general population making it one of the most prevalent chronic inflammatory diseases, leading to debilitating effects which influence patients’ quality of life and their psychological status.^3^ Management of RA aims to sustained clinical remission or low disease activity (LDA). In the past, non-steroid anti-inflammatory drugs, few conventional synthetic anti-rheumatic drugs (csDMARDs), such as methotrexate (MTX), sulfasalazine (SSZ), hydroxychloroquine (HCQ), gold salts and D-penicillamine combined with steroids were the existent therapeutic options.^4,5^ At that time, treatment initiation was delayed, based only on monotherapy. As a result of this approach, many patients had an inadequate response leading to unmet needs for RA treatment.^6,7^ Over the past 25 years, increasingly effective treatments have been continuously developed in the armamentarium of RA management. New csDMARDs, biological (b) and target synthetic (ts) DMARDs have been used to prevent or reduce joint damage, limit or delay radiographic damage progression.^8^ However, unmet needs of RA treatment still exist.^9^ In this context, the American College of Rheumatology (ACR) and the European Alliance of Associations for Rheumatology (EULAR) institutions have released recommendations and guidelines for rheumatologists to help RA patients achieve remission or LDA.^8^ Furthermore, tools for monitoring disease activity have been utilised such as the ACR 20/50/70 clinical response, the disease activity score for 28 joints (DAS-28) and others.^10^ However, despite the above approach and the progress that has been made so far, a significant number of patients do not reach remission or LDA.^9^ On the other hand, combination of csDMARDs appears as effective as biologic agents for most patients with RA.^8,9^ MTX is the “anchor” drug among csDMARDs and can be used as initial therapy or in combination with other csDMARDs or with bDMARDs. In this review, we discuss the usefulness of csDMARDs and combination therapy for RA patients. Thus, we performed a comprehensive search through MEDLINE, EMBASE, Scopus, and Web of Science of the relevant literature from January 1990 to August 2025.

## TREATMENT STRATEGIES

Therapeutic decisions and strategies are very important to obtain a rapid disease control and achieve remission or LDA. Early and correct RA diagnosis and early therapeutic intervention are mandatory. Effective management of inflammation which causes structural damage and disability is crucial. It is also essential to follow the ACR/EULAR recommendations, using the treat-to-target (T2T) strategy.^11^ This comprises four principles: 1) definition of the specific target (remission, LDA), 2) close follow-up and monitoring of disease activity with the use of specific tools (DAS-28, acute phase reactants), 3) treatment adjustment is required in case of failure to achieve the target, 4) shared decision making with the patient.^8,11,12^

Thus, therapeutic intervention starts with MTX, leflunomide (LFN) or SSZ, if MTX is contraindicated or the patient develops drug intolerance. The optimal dose of MTX is 0.2–0.3mg/kg body weight per week, following with folic acid supplementation 5–7mg/week. If after 3 months of MTX therapy, no adequate response is achieved, then the dose of MTX is adjusted to maximum dose ≥25mg/week. After 6 months, if remission or LDA are not reached, then the treatment is modified as follows: 1) in the absence of poor prognostic factors and moderate disease activity, a second csDMARD should be added to MTX, 2) if poor prognostic factors are present such as rheumatoid factor (RF) or/and anti-citrullinated antibodies (ACPA) or/and radiographic erosive changes, moderate to high disease activity, high swollen joint count and increased inflammation markers then b or ts DMARDs should be considered.^8,12^ While the treatment with b or ts DMARDs showed promising and cost-effective results, these agents are more expensive, may increase side effects especially infections (viral, bacterial, opportunistic) and increase the risk of cardiovascular (CV) events.^13^ On the other hand, when starting a therapy with a step-up, or initial combination therapy with csDMARDs, similar results might be achieved.^14–16^ Thus, the arising question is which therapeutic options and strategies are better to use?

### Early RA treatment with combination of csDMARDs

MTX is the “anchor drug” among csDMARDs. It has been used as monotherapy, step-up combination therapy with other csDMARDs, initial combination therapy with csDMARDs, or step-up combination therapy with b or ts DMARDs.^15,16^ MTX plus HCQ or triple therapy of MTX plus HCQ and SSZ are frequently used. Rapid symptom control can be achieved by using steroids (prednisone) as a “bridging” therapy with dose tapering ≤7,5-5mg/day (**Table 1**).^16^ Thus, in everyday clinical practice, early RA intervention starts with MTX as the first choice with or without bridging therapy with prednisone. MTX has shown very good clinical response, an acceptable safety profile and delay of radiographic progression.^16^ Additionally, MTX can be combined with other csDMARDs (**Table 1**).

**Table 1.** Combination therapies of MTX with other DMARDs in RA patients.

**Therapeutic strategies**					
**A**	**B**	**C**	**D**	**E**	**F**
MTX monotherapy plus prednisone*	MTX Plus HCQ ± prednisone*	MTX Plus SSZ ± prednisone*	MTX Plus HCQ+SSZ ± prednisone*	MTX Plus LFN ± prednisone*	MTX Plus bDMARDs ± prednisone*

bDMARDs: biologic DMARDs; DMARDs: disease modifying anti-rheumatic drugs; HCQ: hydroxychloroquine; LFN: leflunomide; MTX: methotrexate; RA: rheumatoid arthritis; SSZ: sulfasalazine;

* prednisone as a bridging therapy; ±: with or without.

The pioneering COmbinatie therapy Bij Rheumatoide Arthritis (COBRA) study, is a double-blind trial of early RA patients, showed that combination therapy was superior to monotherapy as regards the clinical efficacy and long-term structural damage benefit.^17, 18^ Patients were allocated to receive MTX+SSZ plus prednisone 60mg versus SSZ alone, with tapering of prednisone to 7,5mg/day after 6 weeks.^17,18^ Eleven years later, patients on combination therapy appeared to have lower morbidity rates^19^ and after 23 years of follow-up the combination therapy patients had normalised mortality rates, being similar to those of the general population.^20^ Remission was the target of the Finnish Rheumatoid Arthritis combination therapy (FIN-RACo) study. Patients with early RA were allocated to receive combination therapy with MTX+SSZ+HCQ plus prednisone, versus monotherapy (SSZ plus prednisone). Two years later, patients in the combination treatment were in remission as compared to monotherapy. Sustained remission and reduced radiographic damage were observed after 11 years in patients receiving combination therapy.^21,22^ The FIN-RACo study emphasised that early combination with csDMARDs and tight disease control improved long-term radiological outcome in these patients.^23^ In another study, infliximab was added to patients of the FIN-RACo study, for the initial 6 months to investigate if it can improve the 2 year outcome. Both groups were treated with combination therapy and randomised to receive infliximab or placebo. At 24 months both groups were in remission, without significant differences. The addition of infliximab delayed the radiographic progression.^24^ However, after 5 years of follow-up, no differences were observed between the two groups.^25^

The importance of disease tight control led to treat to target (T2T) strategy. The Tight Control of Rheumatoid Arthritis (TICORA) study showed that intensive management with csDMARDs revealed higher remission rates as compared to usual care.^26^ The Behandel Strategien (BeSt) is another tight control multicentre randomised clinical study in early RA patients. Patients assigned in four groups: a) monotherapy with MTX, b) step-up combination therapy using csDMARDs, c) initial combination therapy of MTX plus prednisone, and d) initial combination therapy of MTX plus infliximab. After one year of therapy, patients in groups c and d demonstrated clinical and functional improvement and less radiographic damage, compared to other groups.^27^ After 2 years, 38–48% of patients in all groups were in remission.^28^

The Triple Therapy in Early Active Rheumatoid Arthritis (TEAR-I), is a randomised controlled trial comparing step-up combination therapy with SSZ+MTX+HCQ, versus parallel triple therapy using SSZ+MTX+HCQ. It was showed that step-up therapy is, at least, as effective as the parallel triple therapy.^29^ The TEAR-II is a randomised comparative study of oral triple therapy versus etanercept plus MTX in early RA. Patients were randomised to four groups receiving MTX+etanercept, or MTX+HCQ+SSZ, or step-up MTX+etanercept or step-up MTX+HCQ+SSZ. Triple csDMARDs therapy with MTX and treatment with MTX +etanercept resulted in comparable clinical outcome while, small radiographic differences after 2 years of therapy in favour of MTX+etanercept were noted.^30^ In a double-blind randomised non inferiority study published by O’Dell et al. active RA patients despite MTX treatment were randomised to receive MTX plus SSZ and HCQ, or MTX plus etanercept showing that triple therapy was not inferior to MTX plus etanercept.^31^

The Sweoft trial examined treatment of RA after MTX failure comprising two arms: addition of HCQ and SSZ to MTX, versus addition of infliximab to MTX. After one year, patients receiving infliximab with MTX were clinically superior to those of combination with triple therapy.^32^ However, after 2 years no clinical differences were observed among the groups, while infliximab+MTX had a better radiographic benefit.^33^ Verhoeven et al. in a T2T study, compared tocilizumab monotherapy or combination therapy with MTX versus MTX plus prednisone and observed similar clinical benefits among the treatment groups.^34^

The Care in Early RA (CareRA) study investigated the efficacy of intensive combination strategies in naïve, compared to csDMARDs treated patients. High risk patients were assigned in three groups: the COBRA classic (MTX+ SSZ+ prednisone 60mg/day), COBRA slim (MTX+ prednisone 30mg/day) and COBRA Avant-Garde (MTX+ LFN+ prednisone 30mg/day). Prednisone 60mg was tapered to 7,5mg/day and prednisone 30mg was tapered to 5 mg/day. Remission was achieved in the COBRA classic in 70, 4%, in COBRA slim 73, 6% and in Avant-Garde in 68% patients. It was concluded that MTX with moderate step-down dose of prednisone effectively induced remission in early RA patients.^35^ Furthermore, Verschueren et al., using the COBRA slim scheme, found that MTX+ moderate dose of prednisone is effective, safe, cost-effective and a feasible initial therapy in early RA.^36^ ter Wee et al. examined the non-inferiority of two regimens, the COBRA light (MTX+ prednisolone 30mg) versus original COBRA therapy (MTX+SSZ +prednisolone 60mg) and assessed the effect of adding etanercept. Both treatment strategies had comparable favourable effects as regards disease activity, functional ability and radiographic outcome after one year in early RA patients. The addition of etanercept was often not implemented by physicians and patients receiving it appeared to have limited benefit.^37^ The NORD-STAR (Nordic Rheumatic Diseases Strategy Trials And Registry) study compared the benefits and safety of three biologic agents, with different mode of action, all given in combination with MTX versus active conventional treatment in naïve early RA patients. High risk patients treated with MTX were allocated in: a) active conventional treatment with prednisone 20mg, or SSZ+ HCQ+ intramuscular corticosteroids, b) certolizumab, c) abatacept and tocilizumab. All four groups achieved high remission rates. Active conventional therapy was not inferior to certolizumab and tocilizumab, but not to abatacept due to fewer abatacept discontinuations.^38^ MTX safety and efficacy in combination therapies in early RA patients was analysed in a post-hoc analysis of a randomised trial by Lend et al. The results showed that MTX was well tolerated and had a similar safety profile when used in combination with active conventional treatment, certolizumab or abatacept, but the risk of MTX associated adverse events was higher when used in combination with tocilizumab.^39^

In a subgroup analysis of the NORD-STAR trial investigators assessed the effects of the active conventional arms, a) MTX+ oral prednisone 20mg/day, b) MTX-+SSZ+HCQ and mandatory intra-articular corticosteroids injections on clinical outcomes. After 48 weeks both treatment arms were effective and safe. However, patients on triple therapy and corticosteroid injections had better clinical outcomes, fewer withdrawals and adverse events, but a slight worsening of radiographic changes.^40^ In an open-label extension of a randomised, double-blind placebo-controlled study, Kremer et al. demonstrated that combination of MTX plus LFN was effective.^41^ Combination therapy of MTX+LFN was found to be effective and safe for managing RA patients.^42^

A long-term observational study and real-life experience was carried out from our university centre. Early RA patients were treated using all the available csDMARDs and biologic agents, following the T2T and tight control disease assessment. In this study 324 (66%) of patients received MTX monotherapy or combination with other csDMARDs, plus small doses of prednisone and 175 (34%) were treated with bDMARDs, mostly tumour necrosis factor inhibitors (TNFi) with or without csDMARDs and small doses of prednisone. After 12 years of follow-up, the unmet needs of RA treatment were 20,9%. More specifically, 3,2% of patients received csDMARDs and 17,7% of those treated with bDMARDs had an inadequate response to all treatment options and never reached LDA. This is a reasonable size of unmet needs for RA treatment, considering the long follow-up of 12 years.^43^ Thus, patient-tailored treatment decisions, when MTX fails, are more important than drugs in the management of RA.^44^

## COST-EFFECTIVENESS OF csDMARDs

Most studies including combination therapy with csDMARDs demonstrated clinical benefits and improved outcomes while being also cost-effective in early RA patients.^45–47^ Indeed, the COBRA trial reported lower mean cost per patient after 1 year of follow-up using combination therapy versus monotherapy.^48^

In the Rotterdam Early Arthritis Cohort (tREACH) patients were randomised to receive a) initial triple csDMARDs therapy (ITDT) MTX+SSZ+HCQ with intramuscular steroids, b) ITDT with an oral steroid and c) initial MTX monotherapy with oral steroids (IMM). It was showed that treatment goals with triple therapy (groups a and b) were attained more quickly and were sustained as compared to MTX monotherapy.^49^ To evaluate direct and indirect costs per quality adjusted life year (QALY) in the above study, investigators showed that initial triple therapy with csDMARDs had better worker productivity versus MTX alone.^50^ To evaluate the cost-effectiveness of all four interventions in the TEAR II study including immediate triple therapy (IT), immediate etanercept (IE), step-up triple (ST) and step-up etanercept (SE), Jalal et al. found that IT was highly cost-effective. However, IE was more effective in 5 years. The authors concluded that a substantial reduction in the cost of biologic drugs is required to become cost-effective.^51^ To estimate the incremental cost-effectiveness of infliximab versus conventional treatment in the Swefot study, Eriksson et al. investigated patients refractory to MTX, who were randomised to receive infliximab versus those with SSZ+HCQ. It was found that the addition of infliximab as compared to SSZ+HCQ, was not cost-effective over 21 months of treatment.^52^ In a similar study by Eriksson et al. patients with RA refractory to MTX were randomised to receive additional therapy with infliximab or conventional treatment with SSZ+HCQ. It was showed that the addition of infliximab as compared to combination therapy was not cost-effective over 21 months of willingness to pay levels generally considered acceptable.^53^ To evaluate costs and QALYs of treatment strategies in the BeSt study, von der Hont et al. investigated early RA patients who were randomly allocated to four groups: a) sequential MTX monotherapy, b) step-up combination therapy, c) initial combination of MTX with prednisone, or d) initial combination of MTX+ infliximab. It was shown that the initial combination of MTX+ infliximab resulted in better quality of life than other strategies. However, using the friction cost method to achieve costs, this improvement is generally considered to be too high and initial combination therapy of MTX+ prednisone should be preferred.^54^

The comparison of active therapies trial found triple therapy to be non-inferior to etanercept+MTX in patients with active RA.^31^ Furthermore, Bansbacic et al. examined the cost-effectiveness of MTX+etanercept versus triple therapy (MTX+SSZ+HCQ) as a first line strategy and found that implementing initial biologic therapy without trying triple therapy first, increases costs while also providing minimal incremental benefit.^55^ Patel et al. investigated the cost-effectiveness of combination of csDMARDs versus TNFi in active RA, in a prognostic randomised multicentre trial and concluded that starting treatment with combination of csDMARDs, rather than TNFi achieved similar outcome at lower cost.^56^ A systematic economic analysis review by van der Velde et al, suggests that bDMARDs are not cost-effective compared to csDMARDs for RA in adults.^57^ Although b and tsDMARDs therapy is considered optimal treatment for RA in many developed countries, this is not valid for countries with limited income and resources. Recently Said et al. reported the cost-effectiveness of csDMARDs versus b and tsDMARDs in Zanzibar, Africa. In an economic analysis the authors used a Marcov model to calculate the cost-effectiveness of various csDMARDs strategies in the treatment of RA, for a period of 3 years. Data on costs were obtained from the central medical stores and hospital administration. Treatment strategies were given in sequential approach based on T2T therapy. This included MTX monotherapy, combination of MTX+SSZ+HCQ and MTX followed by one or two b or tsDMARDs. It was found that MTX monotherapy was the cost-effective option for the treatment of RA in this country. Other options may be feasible if the drug prices are lowered, especially for the b and tsDMARDs.^58^

## THE EFFECTS OF CONVENTIONAL THERAPY IN CARDIOVASCULAR RISK

CV manifestations are the most common causes of increased morbidity and mortality in RA patients.^59^ Premature atherosclerosis, coronary heart disease and CV events have been recognised as major determinants of morbidity and mortality in these patients.^60^ Atherosclerosis is a chronic inflammatory process caused by many factors such as hypertension, cigarette smoking, obesity, dyslipidaemia, and physical inactivity.^61^ All the above risk factors occur more frequently in RA patients, as compared to the control group.^62^ Furthermore, atherosclerotic CV events are associated with systemic inflammation, high CRP and ESR, and the burden of RA disease activity.^62–65^ Indeed, RA untreated patients and those with active disease present altered serum lipid profile, expressed by low levels of total cholesterol (TC), low levels of low-density lipoprotein cholesterol (LDL-C) and low levels of high-density lipoprotein cholesterol (HDL-C). This phenomenon is called “lipid paradox” because RA patients with low LDL-C levels still experience CV manifestations.^66^ The exact mechanism for the “lipid paradox” is not well known. However, studies have shown that it is driven by the inflammatory process in RA, which caused increased cholesterol catabolism and alteration of HDL-C antioxidant functions. Indeed, pro- inflammatory cytokines like interleukin-6 (IL-6) and tumour necrosis factor alpha (TNFα) upregulated the LDL receptor on hepatocytes, resulting in an increased update of LDL molecules by the liver and secretion into the bile.^67,68^ Another mechanism is the LDL-C oxidation (ox-LDL-C), by systemic inflammation. These ox-LDL-C molecules, undetected in the serum by the conventional assays, are not metabolised by LDL-C receptors. They are antigenic, causing chronic endothelial cell inflammation and tissue damage.^66^ It is also reported that RA patients with active disease, present high titres of antibodies against ox-LDL-C although the exact mechanism of these antibodies is not well known. In a previous study, anti-ox-LDL antibodies were measured in 140 RA patients, showing a positive correlation with CRP and a negative correlation with HDL-C.^69^

Not only has MTX the potential to reduce inflammation, but also to control synovitis, and slow radiological progression in RA.^15,16^ Furthermore, MTX remains the csDMARD with the strongest evidence of cardio protection, via its anti-inflammatory properties. Over the past three decades several studies have demonstrated an independent association between MTX use and the lower risk of CV events.^70^ Reports by our group showed that in early RA the use of MTX in combination with prednisone promotes elevation in TC and HDL-C levels and decrease the atherogenic ratio TC/HLD-C.^71^ Navarro-Millay et al. evaluated the use of MTX therapy in the lipid profile in RA patients. After 6 months of therapy an increase of TC, LDL-C and HDL-C levels was observed.^72^ Similar results were reported when combination therapy of MTX plus etanercept, or triple therapy of MTX+SSZ+HCQ were used.^73^ MTX has an atheroprotective effect by activating adenosine A2A receptors. It is shown that MTX promotes the facilitation of cholesterol outflow from the foam cells in the arterial atherosclerotic wall.^74^ Indeed, early RA patients treated with MTX showed reduction of intima media thickness (IMT) score in the common medial carotid artery after one year treatment, compared to control groups.^75^ Additionally, patients with RA and active disease present high titters of antibodies against ox-LDL-C, which were decreased after MTX treatment and subsequent control of inflammation.^76^ Furthermore, it has been reported that MTX reduces the risk of CV disease by 21%, myocardial infarction by 18% and may offer protection against atherosclerosis and thrombosis in RA patients.^77^ In a meta-analysis, Sun et al. included 10 studies and 195416 RA patients. The aim was to assess the role of MTX in preventing CV events in RA and in treating patients with coronary disease. It was demonstrated that MTX has a protective effect in development of CV events.^78^ However, in a cohort of patients with documented CV diseases, such as myocardial infarction, ischemic heart disease, along with diabetes mellitus type-2, the use of low-dose MTX did not reduce the numbers of CV events, compared to placebo.^79^ HCQ is another csDMARD used in combination with MTX in the treatment of RA. The use of HCQ has a potential benefit on the atherogenic lipid profile in RA patients, decreasing LDL-C, TC, LDL-C/HDL-C and TC/HDL-C ratios.^80^ Changes in the lipid profile have been reported after the initiation of HCQ in RA.^81,82^ The mechanism underlying HCQ effects on lipid profile is believed to be mediated by solely controlling inflammation. SSZ was associated with significantly lower risk of CV disease. It seems to improve endothelial function and correct dyslipidaemia through inhibition of TNFα, IL-6, and IL-1, and suppresses the activation of nuclear factor kappa-B (NF-kB), a mediator of inflammation.^83^ In a case control study by van Halm et al. it was shown that RA patients who were treated with csDMARDs, especially MTX, had a decreased risk of CV disease in comparison to RA patients who did not receive SSZ, HCQ or MTX.^84^ LFN treatment appears to be associated with reduced CV disease.^85^ However, since LFN causes hyper-cholesterolaemia as side effect in RA patients, monitoring of the lipid profile is necessary.^86^ MTX has the strongest evidence for cardio protection among other csDMARDs, primarily via its anti-inflammatory mechanisms. SSZ and HCQ may confer modest beneficial effects, while LFN demonstrated both favourable and adverse CV signals.^86,87^

## CONCLUSIONS

Biologic therapies have been proved to be highly efficacious for the treatment of RA. With their use it is possible to prevent or reduce joint damage, restrict or delay radiographic damage progression.^8,12^ However, despite the significant progress made in the treatment of RA there are limitations and unmet needs.^88^ In addition, cost-effectiveness of biologic agents is questionable compared to csDMARDs.^57,89^ Furthermore, biologic agents present several adverse events such as infections and CV risk.^13^ On the other hand, several long-term observational studies, reviews, meta-analyses and treatment guidelines recommend a combination of csDMARDs in RA patients, especially in those who fail monotherapy.^90,91^ csDMARDs combination therapy is orally administered, very efficient, safe, and cost-effective.^16^ Furthermore, MTX demonstrated cardioprotective effects and reduced the risk of CV events in RA patients. Unfortunately, csDMARDs combination therapy has not reached wide popularity and acceptance among rheumatologists, in clinical practice. Furthermore, the ACR/EULAR recommendations for RA treatment suggest a csDMARD combination therapy only in patients who fail MTX and have no poor prognostic factors for RA outcome.^8,12^ Although at the time of the introduction of biologics the estimated need was 15–21%,^14,40^ today they are used more frequently. Their use should follow the established recommendations^8,12,92^ and be based on a careful clinical assessment of the patient’s symptoms, after ruling out other conditions and comorbidities that may cause pain and joint stiffness.^93–96^

Although csDMARDs may not be superior to biologic agents, when used early in the treatment of RA, then the effects are similar to those of biologic therapies, at lower cost with reduced CV events. However, RA treatment is individualised considering disease activity, previous therapies, comorbidities, and shared decisions with the patients.

## ARTIFICIAL INTELLIGENCE (AI)-ASSISTED TECHNOLOGY

No Artificial Intelligence (AI) Assisted Technology was used for designing, drafting, or editing this manuscript.
